# The effects of temperature on prevalence of facultative insect heritable symbionts across spatial and seasonal scales

**DOI:** 10.3389/fmicb.2023.1321341

**Published:** 2023-12-08

**Authors:** Marcos Martins, Cássia Siqueira César, Rodrigo Cogni

**Affiliations:** Department of Ecology, University of São Paulo, São Paulo, Brazil

**Keywords:** endosymbiont, temperature, insects, prevalence, induced phenotypes

## Abstract

Facultative inheritable endosymbionts are common and diverse in insects and are often found at intermediate frequencies in insect host populations. The literature assessing the relationship between environment and facultative endosymbiont frequency in natural host populations points to temperature as a major component shaping the interaction. However, a synthesis describing its patterns and mechanistic basis is lacking. This mini-review aims to bridge this gap by, following an evolutionary model, hypothesizing that temperature increases endosymbiont frequencies by modulating key phenotypes mediating the interaction. Field studies mainly present positive correlations between temperature and endosymbiont frequency at spatial and seasonal scales; and unexpectedly, temperature is predominantly negatively correlated with the key phenotypes. Higher temperatures generally reduce the efficiency of maternal transmission, reproductive parasitism, endosymbiont influence on host fitness and the ability to protect against natural enemies. From the endosymbiont perspective alone, higher temperatures reduce titer and both high and low temperatures modulate their ability to promote host physiological acclimation and behavior. It is necessary to promote research programs that integrate field and laboratory approaches to pinpoint which processes are responsible for the temperature correlated patterns of endosymbiont prevalence in natural populations.

## 1 Introduction

Nonessential or facultative inheritable endosymbionts are common and diverse in insects and are often found at intermediate frequencies in natural host populations (Oliver et al., 2014; Smith et al., 2021), prevailing as an intriguing and successful ecological division of the tree of life. Their long-term maintenance are associated with their contrasting acting as parasitic modulators of host’s reproductive system and/or by being mutualistic enhancers of host fitness (Zug and Hammerstein, 2015; Corbin et al., 2017; Renoz et al., 2019). Environmental factors directly affect the strength of the phenotypes induced by symbionts and the efficiency of maternal transmission (Oliver et al., 2014; Corbin et al., 2017; Smith et al., 2021). However, field studies exploring the missing link between the functional ecology of these inheritable microbes with environmental factors remains scarce (Oliver et al., 2014).

An evolutionary model states three key parameters regulating the dynamics and equilibria of facultative endosymbiont frequencies in their host populations: i. the proportion of uninfected individuals produced by infected ones (i.e., strength of imperfect maternal transmission); ii. the fitness of infected individuals relative to the uninfected ones (i.e., strength of mutualistic boost); iii. The costs associated with infection and its transmission phenotypes (i.e., strength of parasitism and parasitic reproductive manipulation; Hoffmann et al., 1990; Hague et al., 2020a). Environmental factors are important selective agents driving all those parameters in nature, hence spatial and temporal variation are expected to reflect different patterns of correlated endosymbiont prevalence in the field. A synthesis on the topic is complex and not yet established. Abiotic factors such as temperature (Hague et al., 2022), humidity and presence of inorganic toxicants (Lemoine et al., 2020), biotic factors such as parasitism and disease (Leclair et al., 2021), host resource (Hague et al., 2020a), competition (Gavotte et al., 2010), and symbiont multiple infections (Toju and Fukatsu, 2011; Zhu et al., 2018) assuredly play a role (Oliver et al., 2014; Heyworth and Ferrari, 2015). Among abiotic factors, temperature might be pivotal in modulating the conditioning for host physiology performance, resource availability and presence and abundance of natural enemies (Leclair et al., 2021), thus affecting endosymbiont titer and host-symbiont interactions (Hague et al., 2020a). Indeed, Corbin et al. (2017) reviews that thermal environments affect endosymbiotic association and its ability to alter host thermotolerance across many species beyond insects, but did not aim to link facultative endosymbiont prevalence patterns to the causative processes. Thus, temperature role remains elusive in shaping facultative endosymbiont success.

This mini-review aims to synthesize the current knowledge and bridge the gap by hypothesizing that temperature increases endosymbiont frequencies by modulating key phenotypes mediating the interaction. Field studies exploring the spatial and temporal variation in endosymbiont frequency related to temperature, as laboratory essays exploring the role of temperature on endosymbiont mediated interaction following the previous evolutionary model are essential sources for a comprehensive view of the phenomena.

## 2 Spatial and seasonal endosymbiont prevalence variation correlates with temperature

The prevalence of facultative inheritable bacteria symbionts in natural insect populations varies in spatial gradients, latitudinal clines and over seasons (Corbin et al., 2017; Carpenter et al., 2021; Smith et al., 2021). Studies on 41 different natural insect populations show spatial or temporal symbiont frequency variation. In 21 of these cases, endosymbiont frequency was positively correlated with temperatures (Table 1). Alternatively, in only 3 of those 41 populations, endosymbiont frequency was negatively correlated with temperature (Table 1). In 17 cases, spatial or temporal symbiont frequency variation was observed, but with no observed correlation with temperature (Table 1). Even though we observed a general pattern of more cases of positive correlations between temperature and endosymbiont prevalence, there was great variation among studies. Some studies report seasonal variation with increases in endosymbiont prevalence in warmer seasons (Carpenter et al., 2021), while other studies report spatial variation with increases in endosymbiont prevalence in warmer regions with lower latitudes and lower altitudes (Zhang et al., 2021). One study predicted higher endosymbiont prevalence from land surface temperature data (Morag et al., 2012). A negative correlation between temperature and endosymbiont prevalence was observed in one study conducted in one site that exhibits extremely high temperatures, showing higher endosymbiont prevalence at milder temperatures (Watts et al., 2009). It is important to note that some studies tested the effect of temperature on the entire endosymbiont community infecting a host and symbiont hitchhiking and gut-microbiota is likely affecting some of the observed patterns (Zhang et al., 2021). As an example, *S. symbiotica* exhibits different patterns of infection in aphids across Belgium that could be related with temperature. However, *S. symbiotica* is found both as an endosymbiont and as a gut-associated bacteria, with plant resources also containing the bacteria, serving as a permanent environmental source of contamination (Pons et al., 2022).

**Table 1:** Evidence of spatial and temporal frequency variation in facultative endosymbiont infections in different groups of insects and its correlation with temperature.

Endosymbiont strain	Host group	Host species	Site	Spatial frequency variation	Temporal frequency variation	Correlation between temperature and endosymbiont frequency	References
*Cardinium*	Diptera	*Culicoides imicola*	Israel	*		Positive	1A,2
	Spider mites	*Tetranychus truncatus*	China	*		-	3B
*Fukatsuia*	Aphids	*Acyrthosiphon pisum*	Pennsylvania USA		*	-	4C
*Hamiltonella defensa*	Aphids	*Acyrthosiphon pisum*	Pennsylvania, Wisconsin and Utah USA	*		-	5,6
			China	*		Positive	7
			Pennsylvania USA		*	-	4C
		*Sitobion avenae*	Central and South-central Chile	*	*	-	8
	Hemiptera	*Bemisia tabaci*	Florida USA	*	*	-	9
*PASS*	Aphids	*Acyrthosiphon pisum*	California USA		*	Positive	10
			Japan	*		Negative	11
*Regiella insecticola*	Aphids	*Acyrthosiphon pisum*	Pennsylvania, Wisconsin and Utah USA	*		-	5,6
			China	*		Positive	7
			Pennsylvania USA		*	-	4C
		*Sitobion avenae*	Central and South-central Chile	*	*	Positive	8
			Nanjing, China		*	Positive	12
*Rickettsia*	Aphids	*Acyrthosiphon pisum*	Japan	*		Positive	11
			China	*		Positive	7
			Pennsylvania USA		*	-	4C
	Hemiptera	*Bemisia tabaci*	Florida USA	*	*	-	9D
	Coleoptera	*Curculio sikkimensis*	Japan	*		Positive	2,13
*Rickettsiella*	Aphids	*Acyrthosiphon pisum*	Pennsylvania, Wisconsin and Utah USA	*		-	5,6
			Pennsylvania USA		*	-	4C
*Serratia symbiotica*	Aphids	*Acyrthosiphon pisum*	Pennsylvania, Wisconsin and Utah USA	*		-	5,6
			China	*		Positive	7
			Pennsylvania USA		*	-	4C
		*Aphis fabae*	Belgium	*		Positive	14E
		*Cinara tujafilina*	Valencia, Spain		*	Positive	15
		*Sitobion avenae*	Nanjing, China		*	Positive	12
*Sodalis*	Coleoptera	*Curculio sikkimensis*	Japan	*		Positive	2,13
*Spiroplasma*	Aphids	*Acyrthosiphon pisum*	Japan	*		-	11
			Pennsylvania USA		*	-	4C
	Coleoptera	*Adalia bipunctata*	Sweden	*		Positive	2,16
*Spiroplasma*	Diptera	*Drosophila hydei*	California, Arizona USA and Sonora, Mexico	*		Negative	17F
		*Drosophila mojavensis*	California, Arizona USA and Sonora, Mexico	*		Negative	17F
	spider mites	*Tetranychus truncatus*	China	*		-	3B
*Wolbachia*	Coleoptera	*Curculio sikkimensis*	Japan	*		Positive	2,13
	Diptera	*Drosophila melanogaster*	Eastern Australia; Eastern North America	*		Positive	18,19
		*Drosophila santomea*	Ilha de São Tomé, West Africa	*		Positive	20,21
		*Drosophila teissieri*	Bioko, West Africa		*	Positive	20,21
		*Drosophila yakuba*	Bioko and Ilha de São Tomé, West Africa	*	*	Positive	20,21
	Spider mites	*Tetranychus truncatus*	China	*		Positive	3B

(A). Land surface temperature data. Prevalence declines with increasing maximum daytime temperature and increases with increasing minimum night-time temperature. (B). A spatial correlation was found for Spiroplasma, Cardinium and Wolbachia, but only Wolbachia was significantly affected by temperature, Spiroplasma and Cardinium were affected by altitude, but not temperature. (C). Did not find significance of temperature impacting endosymbiont dynamics. Although, found a strong effect of hitchhiking (possible confound factor). (D). Temperature does not affect endosymbiont frequency variation, but it affects endosymbiont titer. (E). Extensive field study across Belgium, Italy, France, Germany and Rwanda with many insect species and multitrophic levels. Aphids in Belgium are the main representatives of spatial variation associated with temperature. Aphis fabae the most representative host species. (F). Sites show high to extremely high temperatures, hence no effect of cool temperatures but an optimum at high temperatures. (1). Morag et al., 2012. (2). Corbin et al., 2017. (3). Zhu et al., 2018. (4). Carpenter et al., 2021. (5). Russell et al., 2013. (6). Oliver et al., 2014. (7). Zhang et al., 2021. (8). Sepúlveda et al., 2017. (9). Rossitto De Marchi and Smith, 2020. (10). Montllor et al., 2002. (11). Tsuchida et al., 2002. (12). Liu X.-D. et al., 2019. (13). Toju and Fukatsu, 2011. (14). Pons et al., 2022. (15). Martínez-Díaz et al., 2016. (16). Pastok, 2015. (17). Watts et al., 2009. (18). Kriesner et al., 2016. (19). Hague et al., 2022. (20). Cooper et al., 2017. (21). Hague et al., 2020b.

## 3 Temperature affects the efficiency of maternal transmission

Imperfect maternal transmission might be an important mechanism limiting the prevalence of inheritable symbionts (Oliver et al., 2014). We found 17 studies that showed that temperature affects maternal transmission, expanding studies found in Corbin et al. (2017). In eight studies transmission efficiency was negatively correlated with temperature (Table 2), in five studies transmission efficiency was positively correlated (Table 2), and in one study there was a non-linear correlation between temperature and transmission efficiency (Table 2). Hague et al. (2020b) point temperature as a fundamental determinant on the persistence of *w*Mel-like *Wolbachia* on *D. melanogaster* showing that colder temperatures reduce maternal transmission with its effects on host oocytes providing a cellular basis for the *w*Mel clines observed in Australia (Kriesner et al., 2016; Hague et al., 2022). For instance, both high and low temperature affects the vertical transmission of *Spiroplasma* on the native host *Drosophila nebulosa* (Anbutsu et al., 2008) and *Drosophila hydei* (Osaka et al., 2008). At lower temperatures the density of *Spiroplasma* was reduced as well. Low temperatures are known to cure *Spiroplasma* infections in *D. melanogaster*, whereas impeding higher temperatures might alter either their tissue tropism or motility, thereby resulting in less-efficient vertical transmission (Anbutsu et al., 2008). Liu X.-D. et al. (2019) showed failure in the rate of vertical transmission of *R. insecticola* in aphids at high temperatures. However, when high temperatures are maintained for several generations, the transmission rate of *R. insecticola* is increased by up to 100% (Liu X.-D. et al., 2019). Hence, temperature clearly affects the efficiency of maternal transmission, and this can explain some patterns of spatial variation in symbiont prevalence (Hague et al., 2020a).

**Table 2:** Relationship between phenotype, temperature, and effects on fitness of common facultative endosymbionts.

Phenotype	Endosymbiont	Higher temperature^†^	Lower temperature^†^	Enhanced phenotype	Reduced phenotype	No effect on phenotype	Temperature and phenotype correlation	Numbers of observations	References	Condition
Titer	*Arsenophonus, Rickettsia and Wolbachia*	*			*		Negative	1	1	C
	*Spiroplasma*	*		*			Positive	1	2	A
	*Wolbachia*	*			*		Negative	3	3,4,5	AC
		*		*			Positive	1	6	A
		*		*			Positive	1	7	A
Maternal Transmission	*Wolbachia*	*			*		Negative	1	8	A
			*		*		Positive	1	9	A
		*			*		Negative	4	10–13	A
			*		*		Positive	1	14	AE
		*			*		Negative	1	4	C
	*Spiroplasma*		*		*		Positive	1	15	A
		*	*		*		Non-linear	1	16	A
			*		*		Positive	1	2	A
		*			*		Negative	2	17,18	AC
	*Regiella*		*		*		Positive	1	19	AE
		*			*		Negative	1	20	A
Cytoplasmic incompatibility	*Wolbachia*	*			*		Negative	11	21–30	ABCDE
		*				*	No correlation	4	6,28,31,32	AB
			*	*			Negative	2	24,26	A
	*Cardinium*	*			*		Negative	1	33	B
			*	*			Negative	1	33	B
Male killing	*Wolbachia*	*			*		Negative	3	12,18,34	A
		*				*	No correlation	3	12,18,35	A
	*Spiroplasma*	*			*		Negative	2	16,36	A
		*				*	No correlation	2	15,36	A
			*		*		Positive	3	15,16,37	A
			*			*	No correlation	1	15	A
Feminization	*Wolbachia*	*			*		Negative	1	17	A
		*				*	No correlation	1	17	A
Thelytokous parthenogenesis	*Wolbachia*	*			*		Negative	8	20,38–44	A
			*		*		Positive	2	20,41	A
		*				*	No correlation	1	39	A
			*			*	No correlation	2	44,45	A
Fitness^††^	*Wolbachia*	*				*	No correlation	4	46–49	ABC
		*		*			Positive	3	31,47,49	AC
		*			*		Negative	8	13,30,31,43,48–51	ABCD
			*			*	No correlation	1	48	A
			*	*			Negative	2	48,52	A	
	*Serratia*	*			*		Negative	3	53–55	C
		*		*			Positive	2	54,56	AC
		*				*	No correlation	1	56	A
	*Serratia and Rickettsia*	*			*		Negative	1	53	C
		*		*			Positive	1	56	A
		*				*	No correlation	1	56	A
	*Rickettsia*	*		*			Positive	1	56	A
		*				*	No correlation	2	56,57	AC
		*			*		Negative	1	53	C
	*Regiella*	*			*		Negative	3	54,55,58	C
		*		*			Positive	1	54	C
			*			*	No correlation	1	59	C
	*Cardinium*	*		*			Positive	1	60	A
	*Hamiltonella*	*		*			Positive	2	54,61	BC
		*			*		Negative	6	54,55,58,61–63	ABC
		*				*	No correlation	2	61,64	BC
			*			*	No correlation	1	59	C
			*		*		Positive	1	63	A
	*Hamiltonella and X-type*	*		*			Positive	1	64	C
		*			*		Negative	1	55	C
		*				*	No correlation	1	64	C
	*Hamitonella and Rickettsia*	*			*		Negative	1	63	A
			*			*	No correlation	1	63	A
	*X-type*	*		*			Positive	1	64	C
		*			*		Negative	1	64	C
		*				*	No correlation	1	58	C
Protection against natural enemies	*Spiroplasma*		*		*		Positive	1	65	A
	*Spiroplasma and X-type*	*			*		Negative	1	66	C
	*Hamiltonella*	*			*		Negative	6	55,61,62,67–69	ABC
		*				*	No correlation	1	69	A
			*			*	No correlation	1	69	A
	*Hamiltonella and X-type*	*			*		Negative	1	67	B
		*		*			Positive	1	55	C
	*Wolbachia*	*		*			Positive	1	70	F

A. Constant temperature; B. Varying temperature; C. Heat shock; D. Temperature constantly varying through the day; E. Varying temperature in the field; F. Developmental temperature. (1). Barman et al., 2022. (2). Osaka et al., 2008. (3). Wiwatanaratanabutr and Kittayapong, 2009. (4). Mancini et al., 2021. (5). Moghadam et al., 2018. (6). Mouton et al., 2006. (7). Strunov et al., 2013. (8). Trpis et al., 1981. (9). Perrot-Minnot et al., 1996. (10). Johanowicz and Hoy, 1998. (11). van Opijnen and Breeuwer, 1999. (12). Hurst et al., 2001. (13). Jia et al., 2009. (14). Hague et al., 2020b. (15). Montenegro and Klaczko, 2004. (16). Anbutsu et al., 2008. (17). Sakamoto et al., 2008. (18). Sugimoto et al., 2015. (19). Moran and Dunbar, 2006. (20). Liu X.-D. et al., 2019. (21). Hoffmann et al., 1986. (22). Wright and Wang, 1980. (23). Stevens, 1989. (24). Clancy and Hoffmann, 1998. (25). Nasehi et al., 2022. (26). Bordenstein and Bordenstein, 2011. (27). Ross et al., 2020. (28). Ross and Hoffmann, 2018. (29). Feder and Hofmann, 1999. (30). Ross et al., 2019. (31). Saeed et al., 2018. (32). Ross et al., 2017. (33). Doremus et al., 2019. (34). Hurst et al., 2000. (35). Veneti et al., 2004. (36). Malogolowkin, 1959. (37). Ventura et al., 2014. (38). Tulgetske and Stouthamer, 2012. (39). Pintureau and Bolland, 2001. (40). Pintureau et al., 1999. (41). Zhou et al., 2019. (42). Ning et al., 2019. (43). Girin and Boulétreau, 1995. (44). Xi and Joshi, 2016. (45). Reumer et al., 2012. (46). Harcombe and Hoffmann, 2004. (47). Burdina et al., 2021. (48). Dionysopoulou et al., 2020. (49).Gruntenko et al., 2017. (50). Foo et al., 2019. (51). Min and Benzer, 1997. (52). Reynolds et al., 2003. (53). Montllor et al., 2002. (54). Russell and Moran, 2006. (55). Guay et al., 2009. (56). Chen et al., 2000. (57). Cass et al., 2016. (58). Heyworth et al., 2020. (59). Łukasik et al., 2011. (60). Yang et al., 2021. (61). Higashi et al., 2020. (62). Cayetano and Vorburger, 2013a. (63). Shan et al., 2014. (64). Doremus and Oliver, 2017. (65). Corbin et al., 2021. (66). Heyworth and Ferrari, 2015. (67). Doremus et al., 2018. (68). Bensadia et al., 2006. (69). Cayetano and Vorburger, 2013b. (70).Chrostek et al., 2021.

† Exposed and compared to control temperature.

†† Multiple measures: fecundity, survival, body size and development time.

## 4 Temperature affects reproductive parasitism phenotypes

*Wolbachia, Cardinium* and *Spiroplasma* can manipulate the reproduction of their hosts and temperature can affect the expression of these phenotypes (Corbin et al., 2017). Reproductive parasitism phenotypes include cytoplasmic incompatibility (CI), male killing (MK), feminization and thelytokous parthenogenesis (Werren et al., 2008). All these phenotypes increase the number of infected females in the population, either by modification of gametic compatibility and interference in sex determination as non fertilization and selective lethality to males (Hurst et al., 2000; Sakamoto et al., 2008; Sugimoto and Ishikawa, 2012; Liu Q.-Q. et al., 2019; Kaur et al., 2021). The effect of temperature on reproductive manipulation was reported in 46 cases (Table 2). In 28 cases the expression of the reproductive manipulation was stronger at lower temperatures (negative correlations in Table 2), in five, reproductive manipulation was stronger at higher temperature (positive correlations in Table 2) and 13 cases showed that the expression of reproductive manipulation was not affected by temperature.

Most studies show a pattern that at lower temperatures, *Wolbachia* and *Cardinium* can increase the expression of CI and at higher temperatures the expression of CI decreases or is completely suppressed (Table 2). As development time is slower under colder temperatures, endosymbionts might have enough time to induce full CI in the cells of their hosts, despite the effects on symbiont titer (Bordenstein and Bordenstein, 2011; Doremus et al., 2019). Regarding MK, feminization and thelytoky, at permissive temperatures the expression of these phenotypes is close to 100%. When hosts are exposed to both high or low temperatures, the expression of MK by *Spiroplasma* is greatly reduced (Malogolowkin, 1959; Montenegro and Klaczko, 2004; Anbutsu et al., 2008; Ventura et al., 2014) and exposure to extremely high temperatures reduces the expression of MK by *Wolbachia* (Hurst et al., 2000, 2001). Most studies show that both high and low temperatures reduce the expression of feminization and thelytoky by *Wolbachia* (Girin and Boulétreau, 1995; Pintureau et al., 1999; Ning et al., 2019; Zhou et al., 2019). Furthermore, the time and temperature required to affect phenotype penetrance varies among endosymbiont strains and host species (Pintureau et al., 1999; Corbin et al., 2017). This difference might occur because some strains have higher thermal stress tolerance that endosymbionts face according to their global distribution (Ross et al., 2017). Thus, temperature is an important environmental factor influencing the expression of reproductive parasitism phenotypes in nature.

## 5 Temperature affects endosymbiont influence on host fitness

The presence of symbionts usually affects many components of host fitness, such as fecundity, survival, body size, and development time. These effects are likely to be influenced by temperature. We compiled 57 studies testing the effect of temperature on the effects of symbionts on host fitness (Table 2). In 27 studies there was a negative correlation between temperature and host fitness, indicating that symbionts intensively decrease host fitness at higher temperatures. In 14 studies there was a positive correlation between host fitness and temperature and 16 studies showed no correlation (Table 2). Exposure to thermal stress can negatively affect fitness of insects (Harvey et al., 2020). Across studies that investigated fitness costs on insects associated with endosymbionts under thermal stress, there is a general pattern that higher temperatures causes lower fecundity, survival, body size, and development time (Table 2). Few studies investigated associated costs under low temperatures (Reynolds et al., 2003; Łukasik et al., 2011; Shan et al., 2014; Dionysopoulou et al., 2020). Shan et al. (2014) observed lower fitness associated with exposure to cold in aphids infected with *Hamiltonella*. Interestingly, in some cases, symbionts are capable of providing protection against heat stress, as observed in white flies infected with *Cardinium* (Yang et al., 2021), aphids infected with *Fukatsuia* (Heyworth et al., 2020), and flies infected with *Wolbachia* strain *w*MelPlus (Burdina et al., 2021).

## 6 Temperature affects endosymbiont protection against natural enemies

Several facultative endosymbionts protect its hosts against natural enemies and the outcome of the interactions can vary across different temperature ranges (Doremus and Oliver, 2017). Temperature effect on symbiont protection against natural enemies was reported in 11 studies (Table 2). In seven studies the protection phenotype was negatively correlated with temperature, in two, protection was positively correlated and the remaining two showed no correlation between protection and temperature (Table 2). Protection against parasitoids by *Spiroplasma* in flies ceases to happen at low temperatures (Corbin et al., 2021). *Hamiltonella*’s protection against parasitoid attacks in aphids is severely reduced under high temperature regimes (Bensadia et al., 2006; Guay et al., 2009; Cayetano and Vorburger, 2013a; Doremus et al., 2019; Higashi et al., 2020), but not under colder temperatures (Cayetano and Vorburger, 2013b). Interestingly, *Fukatsuia* (X-type) alone does not confer protection against natural enemies, but it is able to rescue *Hamiltonella*’s protection against parasitoids when aphids are exposed to high temperatures (Guay et al., 2009), but this rescue ability may vary between *Fukatsuia* strains (Doremus and Oliver, 2017). *Spiroplasma* titer is reduced under low temperatures, which can affect toxin production and lipid competition, both factors responsible for protection against parasitoids (Corbin et al., 2021). However, in *Hamiltonella* elevated temperatures do not reduce endosymbiont density in aphids. *Wolbachia* induced antiviral phenotype in *Drosophila melanogaster* depends on developmental temperature being strong at 25°C and abolished at 18°C (Chrostek et al., 2021). Therefore, although symbiont density can be a good indicator of protection against natural enemies for some host-endosymbiont-natural enemy interactions (Martinez et al., 2014; Corbin et al., 2021), its relevance in nature remains unknown (Cogni et al., 2021). The mechanisms behind microbe-mediated protection and its relation to changes in temperature range still need further investigation.

## 7 Temperature affects endosymbiont titer and modulates host physiological acclimation and behavior

From the endosymbiont perspective, temperature challenge may lead, in one extreme, to endosymbiont depletion and in the other to an overproliferation that harms its transmission route through host fitness. Since long-term endosymbiosis success depends on the interests of endosymbionts being aligned with those of their host (Werner et al., 2014; Fisher et al., 2017) titer regulation might be an important trait under selection (Duarte et al., 2021). Titer is often found positively correlated with temperature in experiments held at permissive and constant 18 to 29°C (Reynolds et al., 2003; Osaka et al., 2008; Strunov et al., 2013). However, contrasting patterns are found in experiments involving heat shocks. The white-fly *Bemicia tabaci* harbors several endosymbionts under the genera *Rickettsia, Hamiltonella,* and *Wolbachia*. All of them show reduced titer after exposition to higher temperatures, a pattern not displayed by the obligate endosymbiont *Candidatus* Portiera (Barman et al., 2022). Facultative symbionts were reported to mitigate the effect of heat shock on obligate symbionts, aiding the hosts’ survival (Lemoine et al., 2020) and thus ensuring endosymbiont route to offspring despite the reduced titer. In aphids, for example, heat shock increased the adaptive value of a symbiotic infection, implying that hosting a facultative symbiont was more beneficial under heat stress than under permissive temperatures (Tougeron and Iltis, 2022). This is represented by the endosymbionts’ ability to trigger stress-lowering compounds (e.g., heat shock proteins) that modify hosts thermal tolerance through diverse metabolic and physiological pathways (Burke et al., 2010; Brumin et al., 2011; Wernegreen, 2012; Barman et al., 2022; Iltis et al., 2022).

Deleterious effects of thermal stress on symbiont titers may be diminished via other mechanisms such as gene expression or modulation of the host behavior (Brumin et al., 2011; Truitt et al., 2018; Hague et al., 2020a; Barman et al., 2022). Divergent *Wolbachia* strains were reported to affect host preference to different microclimates. *Drosophila melanogaster* infected with A-group *Wolbachia* strains prefer significantly cooler temperatures relative to uninfected individuals (Hague et al., 2020a). On the other hand, *Drosophila mauritiana* infected with the B-group *w*Mau causes hosts to prefer warmer temperatures. This experiment also shows that host temperature preference does not alter *Wolbachia* titer (Hague et al., 2020a). Therefore, coping with temperature challenge via the symbiont effect on host acclimation and behavior is an important feature evolving that can maintain facultative endosymbiont prevalence.

## 8 Discussion

Temperature promotes an everlasting influence on facultative endosymbionts. In spatial and temporal scales higher frequencies are correlated with higher temperatures, whereas, key phenotypes are found with more negative correlations with temperature. This leads to the following questions: How stable are the patterns of prevalence of facultative endosymbionts in field populations considering different temperature ranges? Are there divergent cost–benefit optima among phenotype intensity, fitness and titer at multiple temperatures? How does this shape and maintain the prevalence? To test the stability of the correlations presented here it is important to describe endosymbiont dynamics with long term resampling (Cogni et al., 2017; Rodrigues and Cogni, 2021) and sampling repeated patterns of correspondent temperature correlations such as clinal and seasonal correspondent variation (Cogni et al., 2015; Rodrigues et al., 2021).

Following the evolutionary model, temperature indeed affects the processes that might explain the prevalence patterns, by acting in the infection itself and directly regulating endosymbiont titer, or by affecting the cost–benefit associated with the infection to the host and regulating the intensity of many endosymbiont induced phenotypes. However, key phenotypes such as maternal transmission, reproductive parasitism, host fitness and protection against natural enemies all show more negative correlations with temperature opposed to the prevalence pattern found in spatial-seasonal scales. This is striking, once the evolutionary model points to intensity of the key phenotypes as an essential mechanism for endosymbionts to reach higher frequencies. This shows that the processes on endosymbiont prevalence and success in nature is indeed complex and need further investigation. Temperature is a strong contender but it did not enact in a predictive way. In some systems, such as maternal transmission intensity modulated by temperature explaining prevalence patterns (Hague et al., 2020a) can be bolstered. However, a generalization of the process in the diversity of endosymbiont insect systems is currently not possible. Thus, it is important to delineate studies that investigate titer and divergent cost–benefit optima among phenotype intensity at multiple temperatures (Reynolds et al., 2003; López-Madrigal and Duarte, 2019; Duarte et al., 2021); as investigate measurements of the reaction norm, rather than only a snapshot of the phenomena with just one set of conditions (Thomas and Blanford, 2003).

In an evolutionary context, it’s harder to demonstrate that laboratory-observed patterns can lead to processes such as frequency-dependent selection in the field (Thomas and Blanford, 2003), but we cannot discard this possibility. Therefore, some important caveats here is that almost all studies on the effect of temperature were set in the laboratory. Protection against natural enemies are frequently reported, but correlation with temperature and this phenotype in the field is currently unknown (Cogni et al., 2021). Another caveat is given to the origin of the populations considered in each study, as a lab-adapted insect population will certainly show different responses to the processes than a wild one. Although many studies reported the conditioning set in their experiments (described in Table 2), the depiction of the events of each considered lab-adapted population is uncertain. If populations were previously acclimated in higher or lower temperatures or were exposed to heat or cold shocks that will assuredly impact on the coevolution processes. An integrative approach combining field and laboratory studies, exploring each system’s field prevalence and laboratory studies including cage experiments exploring temperature variation and realistic upper and lower extremes, as well as constant temperatures is essential. To add another layer, the use of transcriptomics and genomics may help to elucidate possible endosymbiont molecular candidates that interact with hosts that are differentiable between different temperatures, hence directing towards a mechanistic understanding of the patterns.

## Author contributions

MM: Writing – original draft, Writing – review & editing. CC: Writing – original draft, Writing – review & editing. RC: Writing – original draft, Writing – review & editing.
